# *T. brucei* Infection Reduces B Lymphopoiesis in Bone Marrow and Truncates Compensatory Splenic Lymphopoiesis through Transitional B-Cell Apoptosis

**DOI:** 10.1371/journal.ppat.1002089

**Published:** 2011-06-30

**Authors:** Viki Bockstal, Patrick Guirnalda, Guy Caljon, Radhika Goenka, Janice C. Telfer, Deborah Frenkel, Magdalena Radwanska, Stefan Magez, Samuel J. Black

**Affiliations:** 1 Department of Veterinary and Animal Sciences, University of Massachusetts, Amherst, Massachusetts, United States of America; 2 Laboratory for Cellular and Molecular Immunology, Vrije Universiteit Brussel, Brussels, Belgium; 3 Department of Molecular and Cellular Interactions, VIB, Brussels, Belgium; 4 Unit of Veterinary Protozoology, Institute of Tropical Medicine, Antwerp, Belgium; 5 European Cooperation for Science and Technology (COST), Brussels, Belgium; NIAID/NIH, United States of America

## Abstract

African trypanosomes of the *Trypanosoma brucei* species are extracellular protozoan parasites that cause the deadly disease African trypanosomiasis in humans and contribute to the animal counterpart, Nagana. Trypanosome clearance from the bloodstream is mediated by antibodies specific for their Variant Surface Glycoprotein (VSG) coat antigens. However, *T. brucei* infection induces polyclonal B cell activation, B cell clonal exhaustion, sustained depletion of mature splenic Marginal Zone B (MZB) and Follicular B (FoB) cells, and destruction of the B-cell memory compartment. To determine how trypanosome infection compromises the humoral immune defense system we used a C57BL/6 *T. brucei* AnTat 1.1 mouse model and multicolor flow cytometry to document B cell development and maturation during infection. Our results show a more than 95% reduction in B cell precursor numbers from the CLP, pre-pro-B, pro-B, pre-B and immature B cell stages in the bone marrow. In the spleen, *T. brucei* induces extramedullary B lymphopoiesis as evidenced by significant increases in HSC-LMPP, CLP, pre-pro-B, pro-B and pre-B cell populations. However, final B cell maturation is abrogated by infection-induced apoptosis of transitional B cells of both the T1 and T2 populations which is not uniquely dependent on TNF-, Fas-, or prostaglandin-dependent death pathways. Results obtained from ex vivo co-cultures of living bloodstream form trypanosomes and splenocytes demonstrate that trypanosome surface coat-dependent contact with T1/2 B cells triggers their deletion. We conclude that infection-induced and possibly parasite-contact dependent deletion of transitional B cells prevents replenishment of mature B cell compartments during infection thus contributing to a loss of the host's capacity to sustain antibody responses against recurring parasitemic waves.

## Author Summary

African trypanosomiasis caused by *Trypanosoma brucei* species is fatal in both humans and animals and cannot be combated by vaccination because of extensive parasite antigenic variation. Effective trypanosome control and clearance from the bloodstream involves the action of antibodies specific for the parasite's highly diverse variable surface glycoprotein antigens. However, experimental infections in mice have shown that trypanosomiasis elicits a rapid process of B cell exhaustion and loss of protective antibody responses. Indeed, both marginal zone B cells, the first line of defense against blood-borne pathogens like *T. brucei* parasites, and follicular B cells, which are the major source for developing high-affinity antibody-producing plasma cells and memory B cells, become depleted during infection. In addition, existing B-cell memory, both against parasite antigens and non related pathogens, is destroyed early on in infection. Here, we demonstrate that during infection, B cell development is decreased in the bone marrow and early B cell development is taken over by the spleen. However, full maturation of developing B cells is abrogated by the occurrence of transitional B cell apoptosis. This impairs the replenishment of the mature marginal zone and follicular B cell pools and prevents the buildup of protective immunity against successive parasitemic waves.

## Introduction

*Trypanosoma brucei* is a highly antigenically variable uniflagellate protozoan of which the subspecies *T. b. gambiense* and *T. b. rhodesiense* cause Human African Trypanosomiasis (HAT), also called Sleeping Sickness. In addition the parasite infects domestic animals, contributing to Nagana, which is a fatal disease of livestock in sub-Saharan Africa. *T. brucei* is transmitted in tsetse fly saliva and lives and replicates in blood, lymph and interstitial fluids of its mammal hosts protected from lytic plasma components by a coat of variable surface glycoprotein (VSG). The surface coat of a *T. brucei* parasite consists of 10^7^ identical densely packed VSG molecules which can be varied among a possibly unlimited repertoire of coat types via a mechanism called antigenic variation [1]–[5]. Clearance of *T. brucei* and other African trypanosomes from the host blood stream is mainly mediated by VSG specific antibodies [6]–[8]. *T. brucei* parasites have been shown to (i) deplete marginal zone and follicular B cells from the spleen [9] , (ii) induce non-specific, polyclonal B cell activation leading to clonal exhaustion [10]–[12], and (iii) cause a general decrease in bone marrow cells [13] consistent with a negative impact on lymphopoiesis and erythropoiesis. Infection of trypanosomiasis-susceptible hosts with African trypanosomes has been shown to compromise host humoral immune competence resulting in the loss of B cell responsiveness to new antigens and of recall responses to previously encountered antigens, including trypanosome VSGs and vaccines [9]. Hence, vaccination against trypanosomiasis has so far never been successful in a natural infection setting.

B2 B cell lineage development under normal conditions occurs via a series of bone marrow (BM) stromal cell facilitated processes that begin within the hematopoietic stem cell pool and proceed in hierarchical steps of lineage commitment [14], [15]. Hematopoietic stem cells (HSC), which can self renew, give rise to multi lineage progenitors (MLP) and lymphocyte primed multi lineage progenitors (LMPP) that no longer self renew. LMPP, in turn, give rise to common lymphoid progenitors (CLP), which have been shown to sustain both T and B lymphopoiesis, although these lineages may diverge within the CLP. CLP give rise to several types of precursor cells, including pre-pro-B cells [16], [17]. B lymphopoiesis then proceeds in the bone marrow yielding several developmental stages of pre-pro-B, pro-B, pre-B and eventually immature B cells, which show a high expression of the IgM form of the antigen receptor and low or no expression of the IgD maturation marker [18], [19]. To complete their development, immature B cells migrate through the periphery, however only 10% reaches the spleen as transitional B cells of the T1 type. Important is the fact that under inflammatory immune conditions, BM lymphopoiesis is often severely reduced, and is compensated for by a splenic cell differentiation process that involves the same B-cell differentiation steps, referred to as extramedullary lymphopoiesis [20], [21]. Once the transitional T1 stage has been reached, B cells develop further into T2 transitional B cells that in turn can mature into either Marginal Zone B (MZB) cells or Follicular B (FoB) cells [22]. T2 cells can also give rise to T3 transitional B cells, but the latter don't give rise to mature B cells, due to being hyper-responsive to stimulation through their BCR [23]. Each of these populations is distinguished by a unique set of cell surface antigens, allowing monoclonal antibody (mAb) phenotyping by multicolor flow cytometry [24]–[26]. Using this approach, Radwanska *et al*. reported that the splenic MZB and FoB cell populations become rapidly depleted in *T. brucei*-infected mice and do not recover [9]. Since these findings suggest an impaired replacement of mature B-cells during infection, we have now investigated B cell development, maturation and cell death of various B-cell populations in *T. brucei*-infected mice.

B-lymphocyte cell death in the context of inflammation and infection has been attributed in the past to several major mechanisms that include TNF-TNFR1 and Fas/Fas-L induced apoptosis, prostaglandin triggered cell death and BCR-cross-linking in the absence of proper T-cell help [20], [27]–[32]. With respect to African Trypanosomiasis, none of these aspects have been addressed so far, despite the crucial need for an effective B-cell compartment for parasitemia control. In contrast, their role and modulation during intracellular *Trypanosoma cruzi* infections is better documented, showing that: (i) in the absence of TNF-TNFR1 signaling, susceptibility to infection increases and coincides with abnormal B-cell differentiation in secondary lymphoid tissues [33]. (ii) CD95/FasL interaction between B cells can mediate the fratricide of IgG+ B lymphocytes [27], and (iii) myeloid cell-derived prostaglandins contribute to infection-associated apoptosis of immature B cells in a Fas-FasL independent manner [28].

Programmed cell death can be induced by a number of death factors, including Fas-FasL interaction [34], [35] and the TNF-TNF-R1 apoptosis pathway [36], [37]. With respect to the latter, it has been well established that (i) *T. brucei* infections induce severe inflammatory disease in susceptible hosts leading to the excessive production of pro-inflammatory factors including TNF and prostaglandins [38]–[42], and (ii) that excess induction of TNF negatively affects various lymphoid compartments [20]. In contrast to TNF, to date no information on the role of Fas has been reported in a *T. brucei* infection setting. The Fas apoptosis pathway is normally important in both the regulation of the immune response as well as T and B lymphocyte homeostasis [35]. Therefore, Fas-FasL mediated apoptosis plays a critical role in the mechanism for negative selection of B cells [43]–[45] and for the establishment of the B cell repertoire in the memory compartment [46]. Following activation, B cells can rapidly upregulate both Fas and FasL expression [47], [48], but the control of B lymphocyte expansion appears mainly to be regulated by FasL-expressing T cells [47].

Here we investigate the contributions of different mechanisms to *T. brucei-*induced abrogation of B cell development and infection-associated B cell apoptosis. Our results show that following *T. brucei* infection, B lymphopoiesis is truncated in the bone marrow and compensatory extramedullary B lymphopoiesis is induced (but not completed) in the spleen. Splenic B lymphopoiesis up to the stage of immature B cells was triggered but final development was severely limited by apoptosis of transitional B cells, thus preventing replenishment of mature B2 B cells. Despite the pro-inflammatory immune environment induced by experimental *T. brucei* infections, these events occurred independent from TNF-TNF-R1, Fas-Fas-L and prostaglandin-mediated pathways. Interestingly, in an *ex vivo* setting in which naïve or infection-derived splenocytes were co-cultured with living bloodstream form trypanosomes, transitional B-cell apoptosis was only observed when cell-cell contact between lymphocytes and parasites occurred. This observation corroborates the previous findings from Radwanska *et al.* that showed that trypanosomes can induce contact dependent cell death in anti-VSG hybridoma B-cells [49]. Using a Trans-well co-culture system, or a VSG specific Nanobody, *i.e.* a variable heavy chain fragment of a single chain camelid antibody devoid of its Fc part [50], we now show that preventing direct contact between the trypanosome surface coat and transitional B-cells results in an abrogation of infection-induced apoptosis in the *ex vivo* setting.

## Results

### Impaired B lymphopoiesis in *T. brucei* infected mice

**Figure 1:**
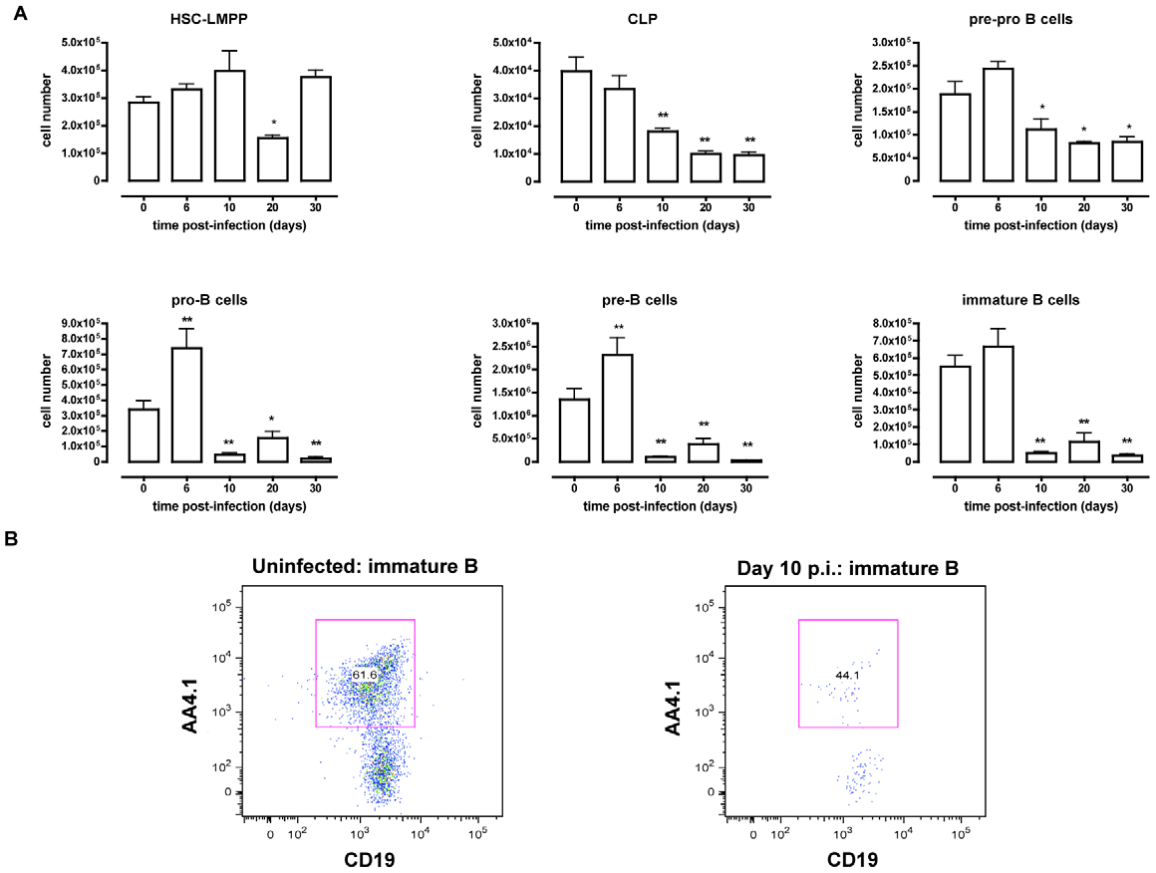
B dyslymphopoiesis in bone marrow during *T. brucei* infection. (A) Bone marrow cells from non-infected mice and mice infected with *T. brucei* for 6–30 days were stained for surface markers commonly used to define developing B cells, as described in table 1 and analyzed using FACS. Data are represented as mean of three mice per group ± SEM, three independent repeat experiments were performed and statistics are compared to uninfected controls (*) p<0,05, (**) p<0,01. (B) Immature B cells were detected as (Lin**^−^**B220^+^IgM^+^CD43^lo/−^ AA4.1^+^CD19^+^) cells in uninfected mice (left panel) versus infected mice on day 10 p.i. (right panel).

*T. brucei* infections in mice have been shown to compromise host humoral immune competence and to induce the loss of specific mature B cell populations in the spleen [9]. However, little is known about how B lymphopoiesis in the bone marrow is affected during *T. brucei* infection. Here, B lymphopoiesis has been examined using a C57BL/6 mouse *T. brucei* AnTat 1.1E infection model, which is characterized by successive waves of parasitemia and a median infection survival time of 35 days [51]. Bone marrow and spleen cells were isolated at different time points of infection and prepared for cellular characterization by multicolor flow cytometry as described in Table 1 and shown in Figure S1, S2 and S3. As presented in Figure 1, the number of very early progenitors, i.e., HSC-LMPP was minimally affected, showing only a transient reduction on day 20 p.i. Subsequent bone marrow B lymphopoiesis was severely affected in *T. brucei* infected mice, with major declines in all bone marrow B cell developmental stages starting with the CLP fraction. A drop in the number of CLP progenitors was detected on day 10 p.i. and this cell population remained severely depleted thereafter. The pre-pro-B cell population showed a 50% reduction by day 20 p.i., while the subsequent B-cell maturation stage *i.e.* the pro-B, pre-B and immature B cell populations reached more than 95% depletion by day 10 p.i. and failed to recover throughout the further course of infection. Combined, these results show that at the end stage of differentiation in the bone marrow, B cell numbers are severely depleted.

**Table 1:** Differentiation antigen phenotypes of developing and mature B2 B cells.

	Suface marker phenotype
**HSC/LMPP**	Lin**^−^** (Ter119, CD3ε, CD11b, Gr1, NK1.1), B220**^−^** ^,^ IL-7r**^−^**, ckit^+^
**CLP**	Lin**^−^** (Ter119, CD3ε, CD11b, Gr1, NK1.1), B220**^−^**, ckit**^−^**, AA4.1^+^, IL-7r^+^
**pre-pro-B**	Lin**^−^** (Ter119, CD3ε, CD11b, Gr1, NK1.1), B220^+^, AA4.1^+^, IgM**^−^**, CD19**^−^**, CD43^hi^
**pro-B**	Lin**^−^** (Ter119, CD3ε, CD11b, Gr1, NK1.1), B220^+^, AA4.1^+^, IgM**^−^**, CD19^+^, CD43^hi^
**pre-B**	Lin**^−^** (Ter119, CD3ε, CD11b, Gr1, NK1.1), B220^+^, AA4.1^+^, IgM**^−^**, CD19^+^, CD43^lo/**−**^
**immature B**	Lin**^−^** (Ter119, CD3ε, CD11b, Gr1, NK1.1), B220^+^, AA4.1^+^, IgM^+^, CD19^+^, CD43^lo/**−**^
**T1 transitional**	B220^+^, AA4.1^+^, IgM^hi^, CD23**^−^**
**T2 transitional**	B220^+^, AA4.1^+^, IgM^hi^, CD23^+^
**T3 transitional**	B220^+^, AA4.1^+^, IgM^lo^, CD23^+^
**MZB**	B220^+^, AA4.1**^−^**, CD1d^hi^
**FoB**	B220^+^, AA4.1**^−^**, CD1d^lo^

B lymphocyte subsets (left column) were identified and defined based on flow cytometric analysis using surface expression of specific antigens (right column).

### *T.brucei* infection induces extramedullary lymphopoiesis in the spleen

**Figure 2:**
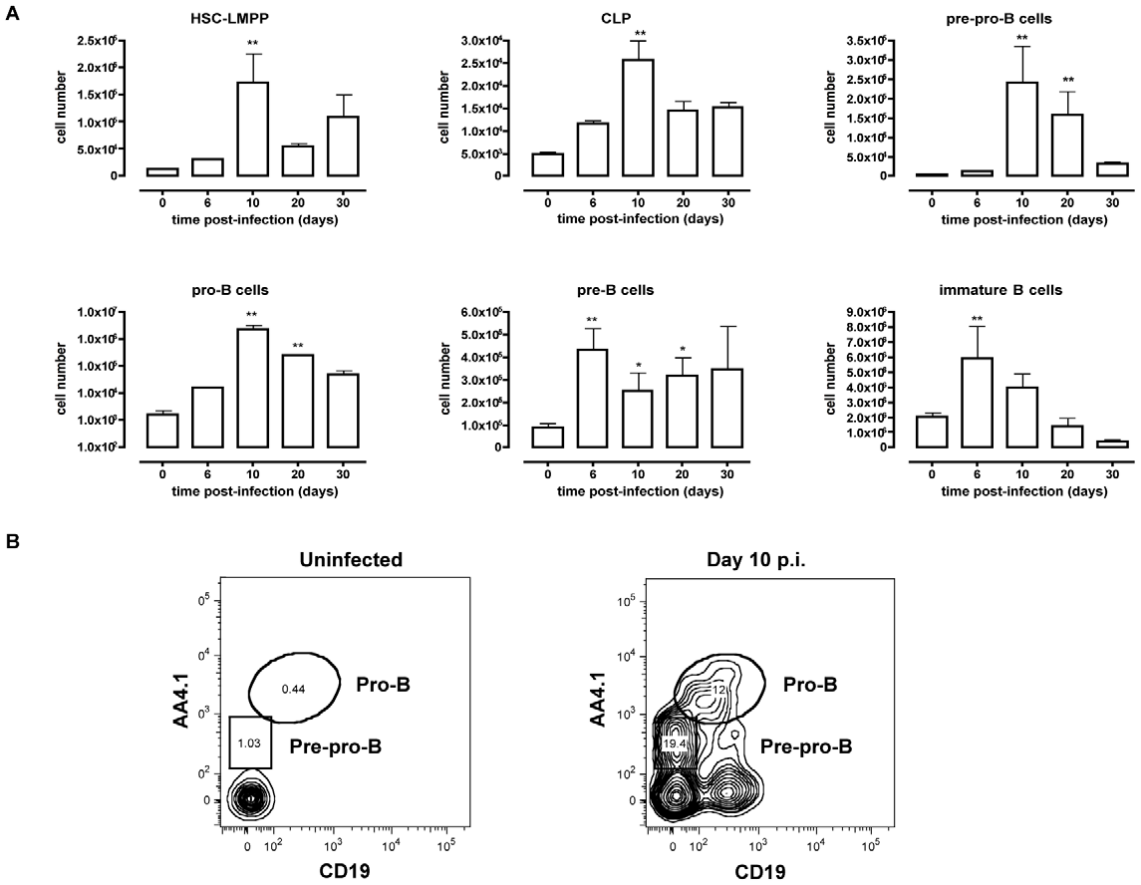
Extramedullary B lymphopoiesis in spleen during *T. brucei* infection. (A) Spleens cells from non-infected mice and mice infected with *T. brucei* for 6–30 days were stained for surface markers commonly used to define developing B cells as described in table 1 and analyzed using FACS. Data are represented as mean of three mice per group ± SEM, two independent repeat experiments were performed and statistics are compared to uninfected controls (*) p<0,05, (**) p<0,01, (***) p<0,001. (B) Pre-pro-B and Pro-B cells were detected as (Lin**^−^**B220^+^IgM^+^CD43^hi^) and respectively AA4.1^+^CD19**^−^** or AA4.1^+^CD19^+^ in uninfected mice (left panel) versus infected mice on day 10 p.i. (right panel).

Inflammation in general has been described to induce mobilization of immature bone marrow lymphocytes [20]. Because *T. brucei* infection is characterized by a strong type 1 inflammatory immune response, mobilization of bone marrow precursors may result in the appearance of developing B cells in the spleen. Cells were harvested from the spleen at different time points of infection and a multicolor flow-cytometric analysis was performed according to Table 1. As shown in Figure 2, *T. brucei* infection induced an increase in HSC-LMPP fractions in the spleen by day 10 p.i. In addition, there was a significant rise in CLP, pre-pro-B, pro-B and pre-B cell numbers in the spleen on day 10 p.i., coinciding with the drastic losses of B cell precursors from the bone marrow. While pre-pro-B, pro-B and pre-B cell numbers remained significantly elevated in the spleen by day 20 p.i., these populations returned to pre-infection levels towards the end of infection (day 30 p.i.). In contrast, while there was no early stage decrease in immature B cells in the spleen, there was a significant loss of this population towards the end of infection.

### *T. brucei* infection causes depletion of transitional B cells in the spleen through induction of apoptosis

**Figure 3:**
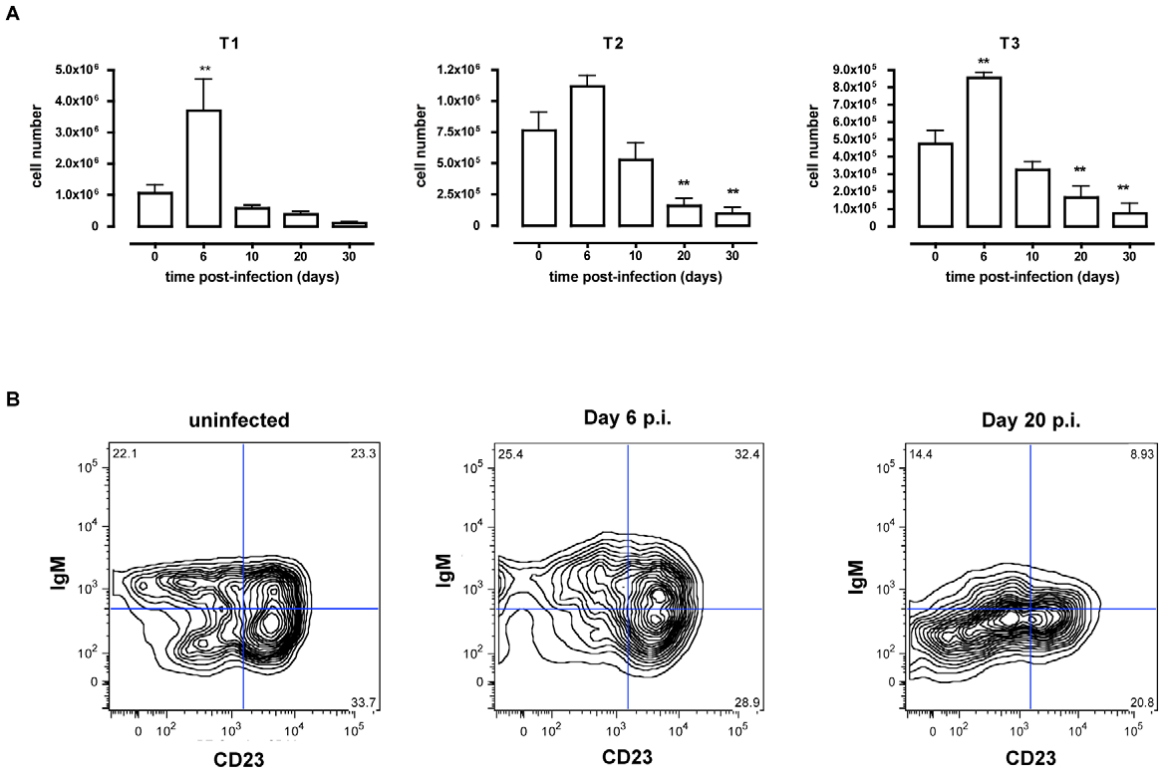
Depletion of transitional B cells in spleen during *T. brucei* infection. (A) Spleens cells from non-infected mice and mice infected with *T. brucei* for 6–30 days were stained for surface markers commonly used to define transitional T1, T2 and T3 B cells and analyzed using FACS. Data are presented as mean of three mice per group ± SEM, three independent repeat experiments were performed and statistics are compared to uninfected controls (**) p<0,01. (B) Transitional T1, T2 and T3 B cells were detected as (AA4.1^+^B220^+^) and respectively IgM^+^CD23**^−^**, IgM^+^CD23^+^ and IgM^lo^CD23^+^ in uninfected mice (left FACS panel) and infected mice on day 6 p.i. (middle FACS panel) and day 20 p.i. (right FACS panel).

In contrast to the elevation in early B cell developmental stages observed in the spleen, Radwanska *et al*. [9] have reported depletion of mature marginal zone and follicular B cells suggesting impaired replacement. Therefore, B cell development at the transitional B cell stage in the spleen was examined here, as these cells provide the link between the immature B cell stage and the mature marginal zone and follicular B cell stages. Flow cytometric analysis of the T1, T2 and T3 transitional B cell populations of *T. brucei* infected mice (Figure 3) revealed a transient increase in transitional B cells numbers that however rapidly faded towards day 10 p.i. On days 20 and 30 p.i. the number of transitional T2 and T3 B cells was significantly decreased compared to uninfected control mice.

**Figure 4:**
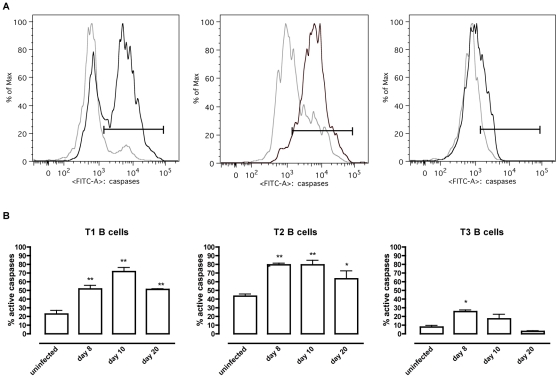
*T. brucei* infection-induced apoptosis of transitional B cells. Spleens cells from uninfected control mice and mice infected with *T. brucei* were stained for surface markers commonly used to define transitional T1, T2 and T3 B cells (as described in table 1) and the amount of active caspases −1, −3, −4, −5, −6, −7, −8 and −9 was measured by intracellular staining using the FAM poly caspases assay kit and flowcytometry. (A) Representative histogram of uninfected controls (grey line) versus a day 10 p.i. (black line). (B) Percentage of apoptotic cells within T1, T2 and T3 transitional B cell populations in uninfected controls versus infected mice on day 8, 10 and 20 p.i. Data are represented as mean of three mice per group ± SEM and two independent repeat experiments were performed (*) p<0,05, (**) p<0,01.

**Figure 5:**
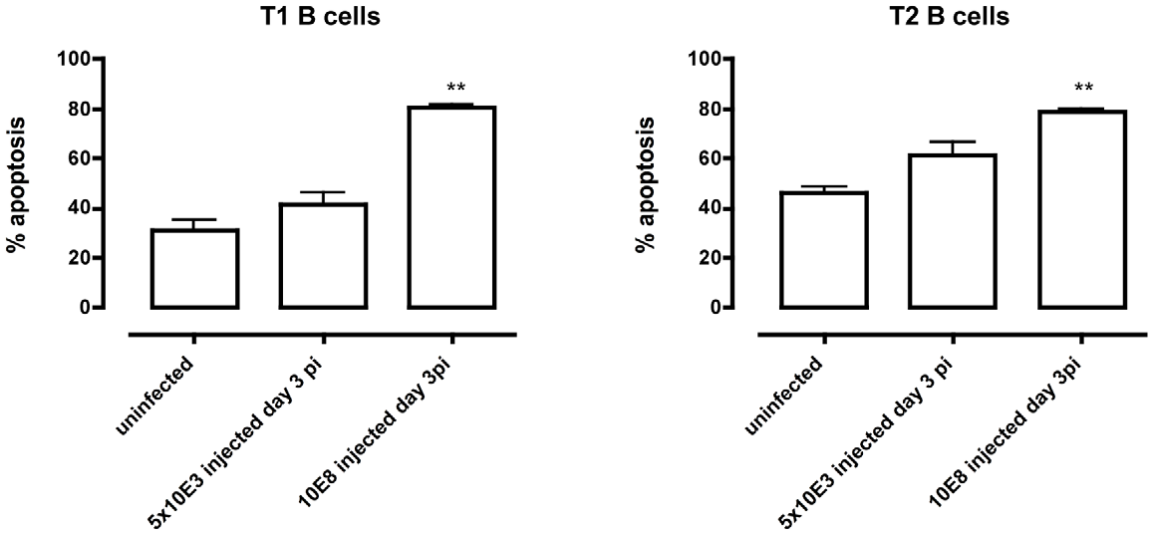
Apoptosis of transitional B cells *in vivo.* Control mice and mice infected with 5×10^3^ or 10^8^ *T. brucei* AnTat 1.1 were killed on day 3 p.i., splenocytes collected and T1 (left) and T2 (right) transitional B cell apoptosis measured. Data are represented as mean of 3 mice ± SEM (**) p<0,01.

To address whether apoptosis is contributing to the depletion of transitional B cells in the spleen, the amount of active caspases-1, −3, −4, −5, −6, −7, −8 and −9 inside the transitional T1, T2 and T3 B cells was measured at different time points of infection by flow cytometry (Figure 4A). Although infection did induce apoptosis in both the T1 and T2 transitional B cell population, the T3 population only showed a temporary increase of caspase activation after the first week of infection (Figure 4B). The elevation in levels of caspases in transitional B cells coincided with the contraction of these B cell populations between days 6 and 10 p.i. (Figure 3A), i.e., immediately following peak parasitemia (8×10^7^ *T. brucei*/ml blood). In the model used here, infections are initiated with 5×10^3^ *T. brucei* AnTat 1.1 and remission of the first parasitemic wave occurs between 6 and 7 days p.i. [41]. It is noteworthy that when the infection was initiated with 10^8^ *T. brucei* AnTat 1.1, peak parasitemia and wave remission occurred at 4 days p.i. (data not shown) and transitional B-cell apoptosis was observed as early as 3 days p.i. (Figure 5). These observations suggest a direct link between levels of parasitemia and the kinetics of induction of splenic T1/2 B-cell apoptosis. Combined, these data indicate the induction of apoptosis in both T1 and T2 transitional B cell populations occurs at, or close to, peak parasitemia. The transitional B cells would, under normal conditions, give rise to the continuous replenishment of marginal zone and follicular B cell populations, but clearly are unable to do so in the infected mice.

### *T. brucei* infection-induced apoptosis of transitional B cells occurs independently of TNF and Fas

**Figure 6:**
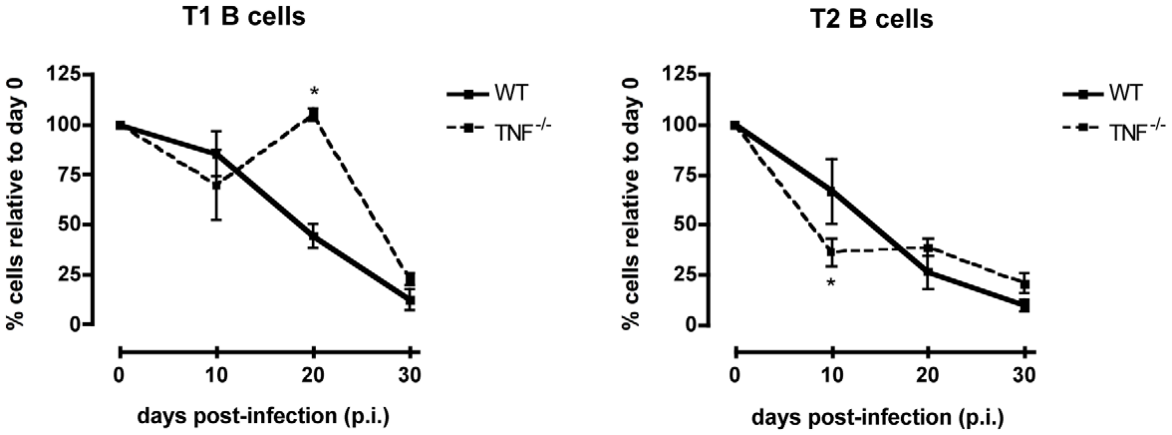
Transitional T1 and T2 B cells in *T. brucei*-infected C57Bl/6 WT versus TNF^−/−^ mice. Percentage of total transitional T1 (left) and T2 (right) B cells in the spleen of uninfected controls (100%) versus *T. brucei*-infected C57BL/6 WT and TNF^−/−^ mice on day 10, 20 and 30 p.i. Data are represented as mean of three mice per group ± SEM and three independent repeat experiments were performed.

Experimental *T. brucei* infections induce high circulating levels of TNF [38], a host cytokine that has been reported to be involved in the induction of immunopathology during *T. brucei* infections [38]–[42]. Furthermore, TNF is known to be a potent inducer of apoptosis through the TNF-R1 signal pathway. Therefore, to examine the possibility of TNF-mediated apoptosis of T1 and T2 transitional B cells, TNF^−/−^ mice were infected with *T. brucei* and the number of T1 and T2 transitional B cells in the spleen was examined at different time points of infection. Figure 6 illustrates that on day 10 p.i. WT mice and TNF^−/−^ mice both suffered from similar levels of transitional T1 and T2 B cell depletion. While on day 20 p.i. a temporary recovery of T1 transitional B cells occurred only in the TNF^−/−^ mice, the same final level of 75% reduction in both T1 and T2 transitional B cells was observed in WT as well as TNF^−/−^ mice towards the end of infection. As an additional control, apoptosis of transitional B cells was recorded using the poly-capase activation FACS analysis outlined above. Here, the percentage of T1 and T2 B cells undergoing apoptosis in *T. brucei*-infected TNF^−/−^ as well as TNF-R1^−/−^ mice equaled the results reported for WT mice (data not shown).

**Figure 7:**
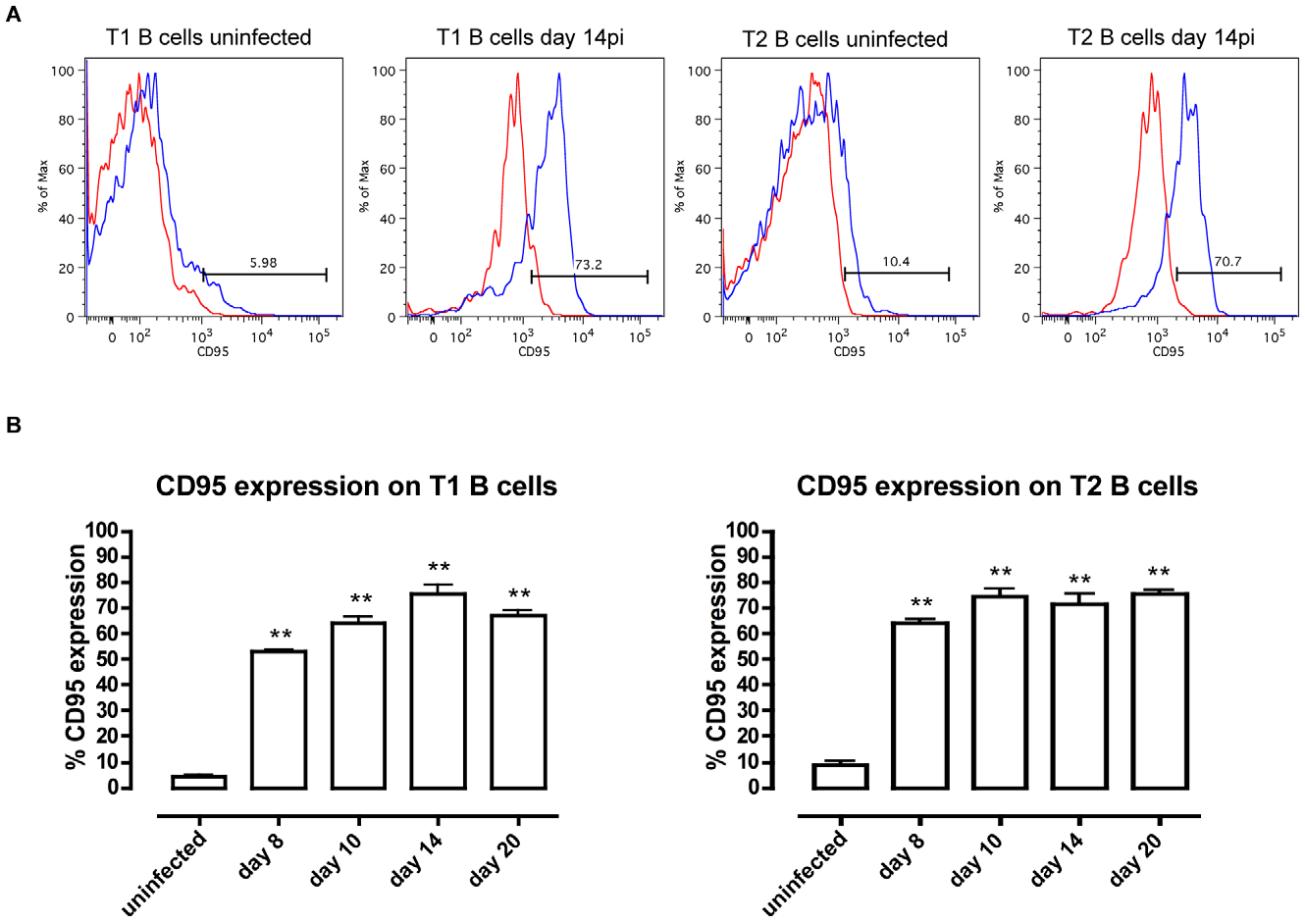
CD95 (Fas) expression on T1 and T2 transitional B cells. (A) CD95 expression (black line) on transitional T1 and T2 B cells versus isotype control (gray line) in uninfected mice and infected mice on day 14 of infection. (B) Percentage of CD95 expression on transitional T1 (left) and T2 (right) B cells in C57BL/6 WT mice on day 8, 10, 14 and 20 p.i. Data are represented as mean of three mice per group ± SEM and two independent repeat experiments were performed (**) p<0,01.

Next, the expression of the death receptor Fas (CD95) on T1 and T2 transitional B cells, and the potential involvement of the Fas-FasL apoptosis pathway were analyzed at different time points of infection. Control T1 and T2 transitional B cells express low, but detectable levels of Fas on their surface. During infection however, there was a strong increase in the level of Fas expression on the surface of both T1 and T2 transitional B cells, as shown here for days 8, 10, 14 and 20 p.i., (Figures 7A and B). The increase in surface Fas expression coincided with elevated caspases activity (Figure 4) and with the loss of transitional B cells from the spleen (Figure 6). Interestingly, also in TNF^−/−^ mice as well as TNF-R1^−/−^ mice, the increase in Fas expression on both T1 and T2 transitional B-cells preceded the rapid loss of these cells from the spleen further suggesting a correlation between the up-regulation of surface-expressed Fas and the trypanosomiasis-associated destruction of the transitional B-cell compartment (Figure S4).

**Figure 8:**
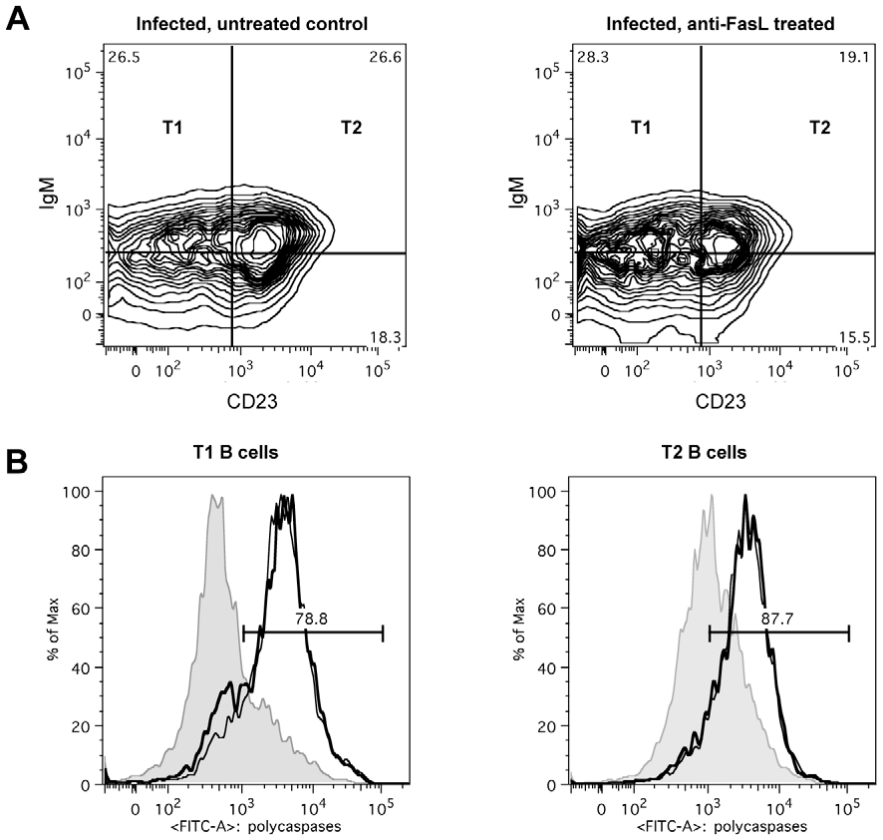
*In vivo* anti-FasL treatment of *T. brucei* infected mice. Mice infected with *T. brucei* were treated with 100 µg of anti-FasL antibody on days 4, 5 and 6 of infection and on day 7 of infection flow cytometric analysis was performed. (A) FACS plot from transitional T1, T2 and T3 B cells, detected as (AA4.1^+^B220^+^) and respectively IgM^+^CD23^−^, IgM^+^CD23^+^ and IgM^lo^CD23^+^ in infected but untreated control mice (left FACS panel), and infected anti-FasL treated mice (right FACS panel). (B) Representative histogram of percentage of apoptotic cells within T1 (left) and T2 (right) transitional B cell populations in uninfected controls (gray area) versus uninfected but treated mice (thin black line) and infected treated mice (thick black line). Data are represented as mean of five mice per group ± SEM.

If apoptosis of transitional B cells in *T. brucei-*infected mice is mediated through Fas, then the loss of the cells would be expected to be reduced in mice with constitutively low Fas expression. However, this proved not to be the case as the extent and kinetics of transitional B cell loss following infection of *lpr* mice with *T. brucei* AnTat 1.1 was the same as in wild type mice (data not shown). In addition, if apoptosis were to be mediated through Fas in wild type mice, it would be expected to be ameliorated by blocking the activation of Fas by its ligand, FasL, through administration of neutralizing anti-FasL antibody [27]. However, this also proved not to be the case. Indeed, mice infected with *T. brucei* and treated by i.v. injection with 100 µg of anti-FasL antibody on days 4, 5 and 6 p.i. did not manifest a measurable alteration in transitional B cell loss from the spleen (Figure 8a), nor did the remaining transitional B-cells in these mice exhibit a change in their caspase activity profile (Figure 8b) relative to mice receiving a control immunoglobulin treatment. Thus, while the occurrence of transitional B cell apoptosis after infection with *T. brucei* coincides with an increased expression of the death receptor Fas on the surface, apoptosis of transitional B cells could not be prevented by the anti-FasL antibody treatment used here.

### Induction of transitional B cell apoptosis occurs independently from the cyclooxygenase pathway

Besides TNF and Fas, also prostaglandins have been implicated in B-cell apoptosis, in particular in a (intracellular) *T. cruzi* infection setting [28]. To examine the possible contributions of cyclo-oxygenase (COX) products to *T. brucei-*induced transitional B cell death *in vivo,* indomethacin (a nonsteroidal anti-inflammatory drug that inhibits COX activity), or control physiological buffer, was administered daily to infected mice by *i.p.* injection using a previously described protocol [52]. The indomethacin treated mice infected with *T. brucei* did not differ from control infected mice with respect to transitional B cell apoptosis (Figure S5). In a second set-up *T. brucei*-infected mice received indomethacin in the drinking water at a concentration of 14 µg/ml [53]. Here again, no difference was observed in the percentage of transitional B cells undergoing apoptosis between treated mice and untreated mice (data not shown).

In addition to the *in vivo* analysis of prostaglandin contribution to the induction of transitional B cell apoptosis, an in vitro assay was designed to further examine the contribution of cyclooxygenase products to transitional B cell apoptosis. Here, spleen cells from uninfected mice were co-cultured in a Trans-well system [29] with spleen cells from uninfected mice or from mice that had been infected for varying times with *T. brucei* Antat 1.1. Culture conditions included incubations in the presence or absence of trypanosomes and of indomethacin. However, in none of the experimental conditions was there a difference in percentage of transitional B cells undergoing apoptosis compared to the controls (representative data are presented in Figure S6), leading us to conclude that prostaglandins (either host or parasite derived) are not a major contributor to transitional B-cell apoptosis during *T. brucei* infections.

### Apoptosis of transitional B cells is blocked when cell-cell contact between trypanosomes and lymphocytes is prevented

**Figure 9:**
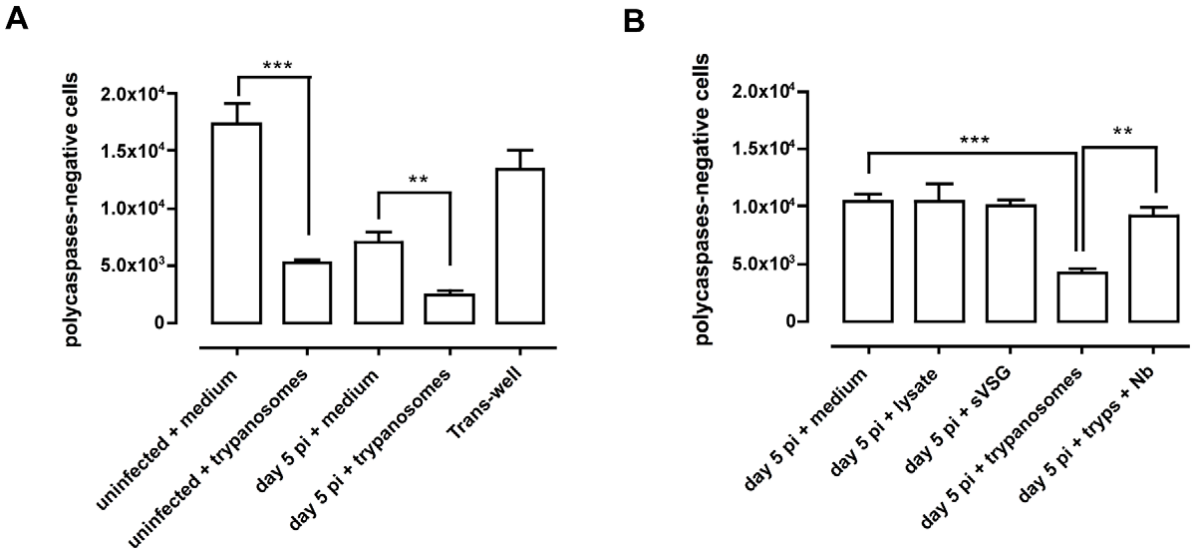
*In vitro* co-culture of total spleen cells and trypanosomes. (A) Total number of viable (or poly caspases-negative) transitional B (T1 and T2) cells left after 20 h of co-culture of total spleen cells from uninfected mice, or mice on day 5 of infection, with live bloodstream form trypanosomes or with medium only as control, or total spleen cells from uninfected mice and trypanosomes separated by a 0,4 µm polycarbonate transmembrane.. Data are presented as mean of three mice per group ± SEM and three independent repeat experiments were performed (**) p<0,01, (***) p<0,001. (B) Total number of viable transitional B (T1 and T2) cells left after 20 h of co-culture of total spleen cells from mice on day 5 of infection with trypanosome lysate, *T. brucei* soluble VSG (sVSG), live bloodstream form trypanosomes, live trypanosomes pre-incubated with VSG-specific nanobodies or only medium as control. Data are presented as mean of three mice per group ± SEM and two independent repeat experiments were performed (**) p<0,01, (***) p<0,001.

When performing the Trans-well experiments described above to address the potential role of prostaglandins in transitional B cell apoptosis, control conditions included co-cultures in which parasites and splenocytes (either from uninfected or day 5 *T. brucei* AnTat 1.1 infected mice) were not separated by a physical barrier. In line with previous results obtained in co-cultures of bloodstream form trypanosomes and B-cell hybridoma cells raised against VSG [49], here the T1/2 transitional B-cell population survival was significantly impaired, which was not observed when parasites and B-cells were separated by a 0,4 µm polycarbonate transmembrane. Indeed, Figure 9A (columns 1 and 2) shows that incubation of spleen cells from uninfected mice with freshly isolated living trypanosomes (10 trypanosome/spleen cell) resulted in 75% lower survival of T1 and T2 B cells relative to incubation in medium. In contrast, there was no difference in the numbers of viable IgM^−ve^ B220^−ve^ leukocytes recovered from splenocytes cultured in the presence or absence of trypanosomes (data not shown).

Similarly, when spleen cells from mice infected 5 days earlier with *T. brucei* were incubated with trypanosomes, there was a 50% reduction in viable T1 and T2 B cells relative to cells cultured in medium (Figure 9A columns 3 and 4) and again no reduction in the recovery of IgM^−ve^ B220^−ve^ leukocytes (data not shown). However, when cultured cells and trypanosomes were separated by a 0.4 µm polycarbonate transmembrane there was no decrease in the number of viable transitional B cells in the culture (Figure 9A column 5) indicating that B cell loss does not result from diffusible trypanosome products or from consumption of essential medium components by living trypanosomes, but from the direct contact between the cells and the parasites. Culturing splenocytes from 5 day-infected mice with parasite lysate, or purified soluble VSG (sVSG) from the parasites, rather than living parasites, also did not result in the loss of transitional B cells (Figure 9B, columns 1–3). To further examine whether contact with VSG on living *T. brucei* is required for depletion of transitional B cells *in vitro*, *T. brucei* AnTat 1.1 trypanosomes were pre-incubated with a VSG-specific Nanobody (Nb, a dromedary heavy chain antibody fragment, devoid of its Fc part, having no detrimental effect on parasite survival [50]), prior to addition to spleen cells from 5 day-infected mice. A comparison of Figure 9B column 4 (control) and column 5 (Nb) shows that the pre-treatment of trypanosomes with nanobodies prevented the killing of B cells. Thus, these results corroborate the obtained Trans-well results indicating that VSG dependent direct contact between intact living trypanosomes and transitional B-cells can trigger cell death.

## Discussion

Infection with African trypanosomes causes mice and other trypanosomiasis-susceptible mammals to develop non-specific hypergammaglobulinemia and polyclonal activation that in the end leads to B cell clonal exhaustion [10]–[12]. While the mechanisms underlying B cell clonal exhaustion have yet to be resolved, results by Radwanska *et al.* showed that the mature marginal zone and follicular B cell populations rapidly disappear during experimental trypanosome infections and that vaccine-induced B-cell memory is destroyed in a non-specific manner [9]. Additional results suggested that while trypanosome infection initially leads to rapid immune activation and buildup of trypanosome/VSG-specific immunity against trypanosomes in the initial parasitemic wave, humoral immune responsiveness is rapidly lost. Impairment in the replacement and recruitment of naïve B-cells to the spleen apparently prevents the efficient activation of specific immunity later on in infection [47], [48]. In order to gain insight into the mechanisms of infection-induced B-cell dysfunction, we have examined how *T. brucei* infection affects B cell development in the bone marrow and the survival and maturation of B cells in the periphery. Our studies show that BM lymphopoiesis is shut down during infection and that compensatory extramedullary B lymphopoiesis is truncated by apoptosis of transitional B cells thus preventing replenishment of mature marginal zone and follicular B cell compartments.

Mice infected with *T. brucei* exhibit reduced numbers of B cells at all developmental stages in the bone marrow and transitional stages in the spleen. Within the bone marrow, loss of CLP preceded that of other progenitor populations raising the possibility that downstream losses resulted from depletion of this precursor population. However, if the loss of CLP was solely responsible for the downstream disrupted B cell development, then the ratio of a subsequent B cell developmental population to its immediate upstream progeny and downstream precursors should remain unaffected, which was not the case. Hence, additional processes besides progenitor depletion must be operating to limit B cell development in *T. brucei* infected mice.

Although aberrant differentiation, apoptosis and expulsion from the bone marrow may singly or jointly contribute to loss of B cell precursors from the bone marrow, we favor the latter mechanisms based on the following arguments: (1) during infection we could not measure any increase in B cell precursor apoptosis in the bone marrow (Figure S7), (2) alterations in the expression of essential B cell development-specific transcription factors in the BM like Icaros, PU.1, EBF and E2A and the IL-7 growth factor have never been reported and were not observed in our laboratory either (data not shown), (3) a reduction in bone marrow CXCL12 expression was the only parameter found by us to correlate with the observed loss of developing B cells during infection (Figure S8). The importance of this result is underlined by the knowledge that inflammation-induced reductions in stromal bone marrow CXCL12 expression indeed have been reported by others to correlate with a premature lymphocyte efflux [20]; (4) there are elevated numbers of HSC-LMPP and CLP as well as pre-pro-B, pro-B, pre-B and immature B cells in the spleen, suggesting the possibility of an increased influx of these cells. In addition, during the early stage of infection small numbers of HSC-LMPP, CLP, pre-pro-B, pro-B, pre-B and immature B cells were also found in the blood, the liver, peritoneum and lungs and small numbers of pro-B, pre-B and immature B cells were found in the lymph nodes. Taking these findings and arguments together, we hypothesize that during early *T. brucei* infections, B cell precursors prematurely migrate out of the bone marrow as a result of the initiation of inflammation, and at least in part home to the spleen, allowing transient extramedullary B lymphopoiesis to take place.

Despite the initiation of extramedullary B lymphopoiesis, infection-induced loss of mature B-cells marks progressing trypanosomiasis in experimental infections. Various factors, in particular the induction of systemic inflammation, could contribute to this. Hence, we addressed the potential involvement of three likely participants, *i.e.* TNF, Fas and prostaglandins, all previously shown to be potentially involved in immune and B-cell malfunctioning. Paradoxically, despite the presence of high systemic TNF levels during infection [38] and the clear evidence shown here that transitional B cell loss through apoptosis coincides with elevation of Fas expression on both T1 and T2 B cells, our results showed through the use of knock-out mice as well as anti-FasL antibodies that neither the TNF- nor the Fas- death pathways acting alone are responsible for transitional B cell apoptosis in a *T. brucei* infection setting. In addition, despite the reported production of prostaglandins by trypanosomes [29] and macrophages in infected mice [54] inhibition of prostaglandin/cyclooxygenase activity in *T. brucei* infected mice by administration of indomethacin did not rescue transitional B cells, contrasting with results obtained in *T. cruzi* infections [28]. However, as each of the death pathways could be redundant in a multi-factorial complex event such as infection-induced apoptosis, their *in vivo* individual contribution to transitional B cell apoptosis in the *T. brucei* infection model should not be formally excluded.

The absence of a clear role for the Fas apoptosis pathway in *T. brucei* induced transitional B-cell death is particularly surprising, taken that (i) Fas upregulation on these cells is reported here to be a clear hallmark of progressing infection, and (ii) that Fas-FasL B cell killing in the context of an infectious disease has previously been described in the case of *Trypansosoma cruzi* infections, which induce Fas-mediated fratricide of IgG^+^ B lymphocytes specific for parasite antigens but not self antigens [27]. In addition, Fas-mediated cell death is known to be important in the regulation of the immune response and T and B lymphocyte homeostasis [35], where for instance Fas-FasL mediated apoptosis plays a critical role in the mechanism for negative selection of B cells [44], [45] and the establishment of the B cell repertoire in the memory compartment [46]. However, despite the reported role for Fas in parasite-induced fratricide, its contribution to the killing of virus-infected, damaged or excess cells and its implication in various immunopathological disorders [43], [44], [47], our study did not provide any evidence functionally linking Fas upregulation and B cell apoptosis. Indeed, treating infected mice three times (with daily interval) with 100 µg of anti-FasL antibody just prior to peak infection, in order to block *in vivo* the activation of the Fas death cascade did not alter the kinetics at which the transitional B cell population underwent apoptosis. It could be argued that the doses of anti-FasL antibody used in this experiment do not functionally block the activation of the Fas apoptosis pathway, however, when *Lpr* mice are infected with *T. brucei*, transitional B cells are lost from the spleen to the same extend as in the wild type mice (data not shown), arguing once again against a major involvement of the Fas-apoptosis pathway.

Having shown that *T. brucei* driven transitional B-cell apoptosis occurs in a TNF-, Fas- and prostaglandin-independent manner, our study next focused on a model system that could help to functionally unravel infection-induced B-cell apoptosis. Interestingly, since B cell apoptosis only occurs when living parasites are administered to the mice, and not when mice are treated on a daily basis by the injection of high doses of trypanosome lysate (results not shown), the presence of a toxin or a super-antigen-like activity by trypanosome molecules appears to be excluded. These results are in sharp contrast to the superantigen-mediated death of mature B cells in the case of *Staphylococcus aureus*, where injection with the virulence factor protein A alone is enough to mimic massive Fas and TNF-independent bacterial induced B cell death, and cause a ‘hole’ in the immune repertoire recognizing the pathogen [55], [56]. Also, it is worth recalling that the *in vivo* experiments presented in this study show that initiation of transitional B cell apoptosis depends on the timing at which peak levels of parasitemia are reached, which is a function of the number of living bloodstream form parasites used for initiation of the infection. This observation suggests a link between the presence of high numbers of living parasites and the induction of parasite-induced transitional B-cell apoptosis consistent with the possibility that B cell hyper-stimulation, through multiple VSG (variable surface glycoprotein - attached to the parasite surface)/BCR (B cell antigen specific receptor - attached to the B-cell surface) interactions, could be a major contributor to this process, as might exposure of B cells to short-lived parasite products which would be active only when directly delivered to the target cell. With respect to BCR signaling, cross-linking of the BCR has been well described to trigger apoptosis of T1 and T2 transitional B cells, which can be ameliorated in the case of T2 B cells by anti-apototic signaling through the BlyS receptor BR3. Transitional B cells of the T2 type can also be rescued from BCR crosslinking-induced apoptosis by T cell help [30]–[32], [57], [58]. However, T cell suppression is one of the hallmarks of *T. brucei* infection [54], making it likely that T2 transitional B cells in infected mice are exposed to the parasites in the absence of proper survival-stimulating T cell help. Of crucial importance is the notion that membrane-bound antigens that can extensively engage BCRs trigger rapid BCR-mediated apoptosis in a Fas-independent manner [59]. Hence, in the context of trypanosome-B cell interaction, the presence of 10 million identical VSG molecules densely packed on the surface of the parasite could be responsible for causing BCR receptor clustering on the surface of the transitional B cells, leading to hyper-stimulation of the B cell and TNF/Fas-independent cell death in the absence of proper T-cell signaling. Avidity, due to multiple VSG/BCR interactions, in this case would have a much higher impact than actual BCR affinity/specificity for a given VSG.

Using an *in vitro* co-culture system of splenocytes and live bloodstream form trypanosomes, we showed that direct contact between the living parasites and host B cells can indeed trigger the deletion of the latter. Depletion of T1 and T2 transitional B cells was maximally induced with 10 trypanosomes per spleen cell in culture but was observed with as few as 0.1 trypanosomes/spleen cell in culture, with 50% depletion occurring at a ratio of between 0.1 and 0.5 trypanosome/spleen cell (Figure S9). This falls within the physiologic range *in vivo* for infections with *T. brucei* AnTat 1.1, where the ratio of trypanosomes to viable nucleated cells in the spleen is 0.25:1 at peak parasitemia. In co-cultures of trypanosomes and splenocytes in which contact between the two is prevented using a Trans-well system or a pre-incubation of the parasite with a VSG-specific Nanobody, abrogation of transitional B cell deletion is observed. This result mirrors a previous observation by Radwanska *et al.* where cell death was triggered in IgM expressing hybridoma cells when cultured in the presence of living trypanosomes [49]. Again, in the co-culture system neither lysate, nor purified parasite VSG could mimic the apoptosis-inducing effect of living parasites (nor could anti-FasL or prostaglandin inhibition block the detrimental effect of living trypanosomes). Together, these observations strengthen the hypothesis that the direct interaction of B cells with epitopes on *T. brucei* causes transitional B cell death. Unfortunately we cannot evaluate Nanobody blockage of transitional B cell apoptosis *in vivo* as these antibody fragments are very rapidly cleared from the circulation by the kidneys [60]. Thus, although the mechanism of *T. brucei*-induced transitional B cell depletion *in vivo* remains to be fully elucidated, we did observe that living trypanosomes induce cell death in transitional B cells *in vitro* through a contact-dependent mechanism. Micro-array analysis of material from both hybridoma co-culture assays and spleen-derived B-cell co-cultures with trypanosomes are now underway in order to shed light on the signal cascades involved in this contact triggered apoptosis.

Combined, our study provides evidence for trypanosomiasis-induced apoptosis of transitional B cells in the spleen and it proposes a mechanism for *T. brucei*-induced B cell clonal exhaustion and loss of humoral immune competence in trypanosomaisis-susceptible hosts. Under normal conditions the production of high-affinity, antigen-specific, class-switched, antibodies takes up to 10 days after immunization [61], [62]. Here, in our infection model, about 90% of the transitional B cells are undergoing apoptosis by day 8 of infection, making the replenishment of the mature marginal zone and follicular B cell populations impossible and therefore obstructing efficient germinal center reaction and the renewal of the plasma B cell pool. Since parasite-specific antibodies are essential for parasite control, inhibition of B cell maturation at the transitional stage is an efficient evasive mechanism developed by the parasite to interfere with the protective antibody responses of the host and establish a sustained infection. It is important to stress here that our studies are based on a mouse model system for African trypanosomiasis, which manifests certain limitations. However, the results obtained in this model provide guidelines for analysis of B cell pathology in more relevant hosts including susceptible livestock species such as cattle and goat, or the retention of immune function in natural trypanotolerant animals such as the Cape Buffalo, which have a sustained capacity to generate effective protective antibody responses against *T. brucei* and other African trypanosomes during chronic infection, thus, maintaining cryptic parasitemia with few or no signs of disease [7]. Based on our findings and the earlier data reported by Radwanska *et al.* [9], it would also be crucial to investigate whether a similar B cell pathology occurs in *T. brucei* infected humans. In this context, it would be interesting to compare B cell pathology between *T. brucei gambiense* (manifesting a chronic infection in humans with prolonged parasitemia control) and *T. brucei rhodesiense* (manifesting an acute infection whereby parasitemia control is lost very early) infected patients, where in the latter it could be that rapid destruction of the B-cell compartment is the underlying reason for failure of parasitemia control, the rapid induction of systemic inflammation, and the subsequent passage of the parasite through the blood-brain barrier.

## Materials and Methods

### Ethics statement

The study was carried out in strict accordance with the recommendations in the Guide for the Care and Use of Laboratory Animals of the National Institutes of Health and Guidelines for the Use of Laboratory Animals in Research, Teaching and Testing of the International Council for Laboratory Animal Science. All animal work was approved by the appropriate committee at the University of Massachusetts (IACUC protocol #s 26-09-09/27-09-09 and 2010-0028) and at the Vrije Universiteit Brussel (ethics committee protocol # 09-220-1).

### Parasites and infection in mice

All mice were housed under barrier conditions. Male C57BL/6 wild type (Taconic, Germantown, NY), *Lpr* and TNF^−/−^ C57BL/6 mice (provided by VUB, Belgium) (7–9 week old) were infected by intraperitoneal (i.p.) injection of 5000 exponentially growing pleomorphic *Trypanosoma brucei* Antat 1.1 (EATRO 1125 stock) [51]. Parasitemia was assessed in blood collected from the tail vein during infection. Blood was diluted in RPMI (Gibco, Grand Island, NY, USA) and the number of trypanosomes present in the blood was estimated using a hemocytometer and a light microscope.

### Cell isolation and flowcytometric analysis

B cell populations were analyzed by flowcytometry. Both spleen and bone marrow from femur and tibia were harvested from non-infected control and infected mice at different time points of infection. Cell suspensions were prepared in FACS buffer (1.0% BSA [Sigma, St. Louis, MO] in DPBS) and red blood cells were lysed using ACK lysis buffer (0.15M NH4Cl, 1.0 mM KHCO3, 0.1 mM Na2-EDTA). Non-specific binding sites were blocked using Fc block (CD16/CD32 Fcγ III/II, BD biosciences, San Jose, CA) for 30 minutes at 4°C. Cells were washed twice with FACS buffer and stained with biotin- or fluorochrome-conjugated primary antibodies (see following section) for 30 minutes at 4°C. After washing twice, cell suspensions stained with biotin-conjugated antibodies were incubated with streptavidin-conjugated fluorochromes (listed in the text), which detects cell bound biotinylated antibodies, and incubated for an additional 30 minutes at 4°C. Finally, cells were resuspended in FACS buffer with 1 µg of 7-amino-actinomycin D (7AAD), a fluorescent DNA dye that binds to membrane permeable dead or dying cells, (BD biosciences). Analyses were performed using a FACS Canto II flow cytometer (BD Biosciences) and data were processed using FLOWJO software (Tree Star Inc., Ashland, OR). The total number of cells in each population was determined by multiplying the percentages of subsets within a series of marker negative or positive gates by the total cell number determined for each tissue.

### Antibodies and detection reagents

The following antibodies were added to 100 µl aliquots of 10^6^ Fc-blocked leukocytes prepared as described above: 0.5 µg anti-IL7rα-FITC (clone A7R34), 0.2 µg anti-IgM-PE (clone II/41), 0.2 µg anti-IgM PE-Cy7 (clone II/41), 0,25 µg hamster IgG2 κ isotype control (clone B81-3), 0.5 µg anti-CD11b-FITC (clone M1/70; 0.5 mg/ml), 0.5 µg anti-CD23-FITC (clone B3B4), 0.5 µg anti-CD45R (B220)-FITC (clone RA3-6B2), 0.2 µg of anti-CD45R (B220)-PE-Cy7 (clone RA3-6B2), 0.2 µg anti-CD93–PE (clone AA4.1), 0.2 µg of anti-CD93-APC (clone AA4.1), 0.5 µg anti-CD95 –FITC (clone Jo2), 0.2 µg of anti-CD117 (ckit)-APC (clone 2B8), purchased from eBioscience (San Diego, CA); 0.2 µg anti-CD1d-PE (clone 1b1), 0.2 µg of anti-CD19-APC-Cy7 (clone 1D3), 0.2 µg of anti-CD43-PE (clone 1B11), 0.2 µg of anti-CD45R (B220)-APC-Cy7 (clone RA3-6B2), 0.2 µg of streptavidin-PerCP, 0.2 µg of streptavidin-PE-Texas Red, purchased from BD Biosciences (Erembodegem, Belgium); 2 µg of each of the following antibodies: CD3ε, CD11b (Mac-1), Gr-1 (Ly-6G and Ly-6C) and Ter-119 (Ly-76) from the Biotin-conjugated Mouse Lineage Panel (BD Biosciences, San Jose, CA).

### Flow cytometric analyses of apoptosis

Cells were stained as described in the previous section with antibodies. For the polycaspases-based apoptosis assay, labeled cells were further reacted with the FLICA^TM^ fluorescent inhibitor of caspase-1, −3, −4, −5, −6, −7, −8 and −9, using the FAM Poly Caspases Assay Kit for flow-cytometric analysis (Molecular probes, Invitrogen, Leiden, the Netherlands).

### In vivo anti-FasL treatment

Mice infected with *T. brucei* where treated i.v. with 100 µg of purified anti-FasL antibody (clone MFL3, purchased from BD Biosciences) on days 4, 5 and 6 of infection and on day 7 of infection total spleen cells were isolated from treated mice and untreated controls and prepared for flowcytometric analysis as described above.

### Co-culture experiments with total spleen cells and live trypanosomes

On day 5 of infection mice were sacrificed to collect blood. In addition, total spleen cells were isolated from the infected mice and uninfected control mice and prepared for cell culture as described above. Trypanosomes were purified from the blood by anion exchange chromatography according to the method of Lanham & Godfrey [63]. Then, 10^6^ total spleen cells were put in co-culture with or without 10^7^ (or fewer) live bloodstream form parasites at 37°C, 5% CO2 and 95% humidity in RPMI1640 medium containing 10% FBS, 2 mM pyruvate, 0.2 mM 2-mercaptoethanol, and penicillin/streptomycin under different experimental conditions and 20 h later, the cells were stained for flowcytometric analysis. Parasite lysate was prepared by 3 repeated cycles of freezing at −80°C and thawing and used at indicated concentration. Soluble VSG (sVSG) was prepared through heat-shock treatment of a purified trypanosome suspension, which forces them to release their VSG molecules, followed by anion exchange chromatography, and used at indicated concentrations. Nanobodies against soluble trypanosome VSG were prepared as described in [50], starting from an immune library of VHH fragments of heavy chain dromedary antibodies, obtained after multiple vaccination with *T. brucei* AnTat 1.1 VSG. Trypanosomes were pre-incubated with Nanobody BankIt1413802 Nb_An05–04 HQ680968 (monovalent and devoid of an Fc chain; 15 µg/ml medium ) at the indicated concentrations 1 hour prior to addition to the splenocytes, and the antibody was included in culture medium during subsequent incubations. Under these assay conditions, binding of the Nanobody onto the trypanosome surface did not result in altered parasite survival.

### Statistical analysis

Statistical comparisons were performed by ANOVA and means were compared using Tukey and Dunnett's post test when p≤0.05 (GraphPad Prism v.4.0, GraphPad Software Inc. San Diego, CA).

## Supporting Information

Figure S1 **HSC/LMPP and CLP gating strategy.** Representative plots obtained using bone marrow or spleen cells from uninfected mice stained for HSC/LMPP and CLP cells as described in table 1.
(TIF)

Figure S2 **Developing and immature B cell gating strategy.** Representative plots obtained using bone marrow or spleen cells from uninfected mice stained for pre-pro B, pro-B, pre-B and immature B cells as described in table 1.
(TIF)

Figure S3 **Transitional and splenic mature B2 B cell gating strategy.** Representative plots obtained using spleen cells from uninfected mice stained for Transitional T1, T2 and T3 B cells and mature MZB and FoB cells as described in table 1.
(TIF)

Figure S4 **CD95 (Fas) expression on T1 and T2 transitional B cells in TNF^−/−^ and TNF-R1^−/−^ mice.** (A) Percentage of CD95 expression on transitional T1 (left) and T2 (right) B cells in C57Bl/6 WT mice versus TNF**^−^** ^/**−**^ mice in uninfected controls and on day 14 pi. (B) Percentage of CD95 expression on transitional T1 (left) and T2 (right) B cells in C57Bl/6 WT mice versus TNF-R1**^−^** ^/**−**^ mice in uninfected controls and on day 14 pi. Data are represented as mean of three mice per group ± SEM.
(TIF)

Figure S5 ***In vivo*** **inhibition of the cyclooxygenase pathway during** ***T. brucei*** **infection using indomethacin,** Indomethacin was administered to mice infected with *T. brucei* and uninfected control mice by daily *i.p.* injection and on day 10 of infection mice were sacrificed and an apoptosis assay was performed. (A) Representative histogram of the amount of active caspases inside T1 transitional B cells (upper panel) and T2 transitional B cells (lower panel). (B) Percentage of transitional T1 (left) and T2 (right) transitional B cells undergoing apoptosis. Data are represented as mean of 2 mice per control group and 3 mice per experimental group ± SEM.
(TIF)

Figure S6 ***In vitro*** **co-culture transwell system to investigate the contribution of the cyclooxygenase (COX) pathway in the induction of transitional B cell apoptosis.** Total spleen cells from uninfected mice were co-cultured in a transwell system separated by a 0,4 µm polycarbonate transmembrane, with either medium or uninfected cells as a control or total spleen cells from mice 5 days post infection in the absence or presence of indomethacin (a nonsteroidal anti-inflammatory drug that inhibits COX activity) and/or trypanosomes. (A) Representative plots of T1 (AA4.1^+^ B220^+^ IgM^hi^ CD23**^−^**) and T2 (AA4.1^+^ B220^+^ IgM^hi^ CD23^+^) transitional B cells cultured under the different conditions. (B) Percentage of transitional B cells undergoing apoptosis in the different co-culture conditions.
(TIF)

Figure S7 **Percentage of developing B cell apoptosis in bone marrow during** ***T. brucei*** **infection.** Bone marrow cells from non-infected mice and mice infected with *T. brucei* for 6–10 days were stained for surface markers commonly used to define pre-pro-, pro- and pre-B cells (left) and immature B cells (right), as described in table 1 and consecutively stained for flow cytometric apoptosis detection by binding of Annexin V. Data are represented as mean of three mice per group ± SEM, two independent repeat experiments were performed.
(TIF)

Figure S8 **Bone marrow CXCL12 mRNA expression during** ***T. brucei*** **infection** BM was isolated and amplified using intron-spanning primers specific for CXCL12 via Quantitative PCR. Data were normalized to GAPDH expression and are presented as relative expression compared to uninfected controls. Data are represented as mean of 6 or 8 mice ± SEM. (*) p<0,05, (**) p<0,01.
(TIF)

Figure S9 *I* ***n vitro*** **co-culture of total spleen cells and trypanosomes.** Total number of poly caspases-negative transitional B (T1 and T2) cells left after 20 h of co-culture of total spleen cells from uninfected mice with live bloodstream form trypanosomes or with medium only as control. Data are presented as mean of three mice per group ± SEM and three independent repeat experiments were performed (**) p<0,01.
(TIF)
