# Supplementary figures and images for: T. brucei Infection Reduces B Lymphopoiesis in Bone Marrow and Truncates Compensatory Splenic Lymphopoiesis through Transitional B-Cell Apoptosis
Source: PLoS Pathog. 2011 Jun 30;7(6):e1002089. doi: 10.1371/journal.ppat.1002089 (PMC3128123; doi:10.1371/journal.ppat.1002089)

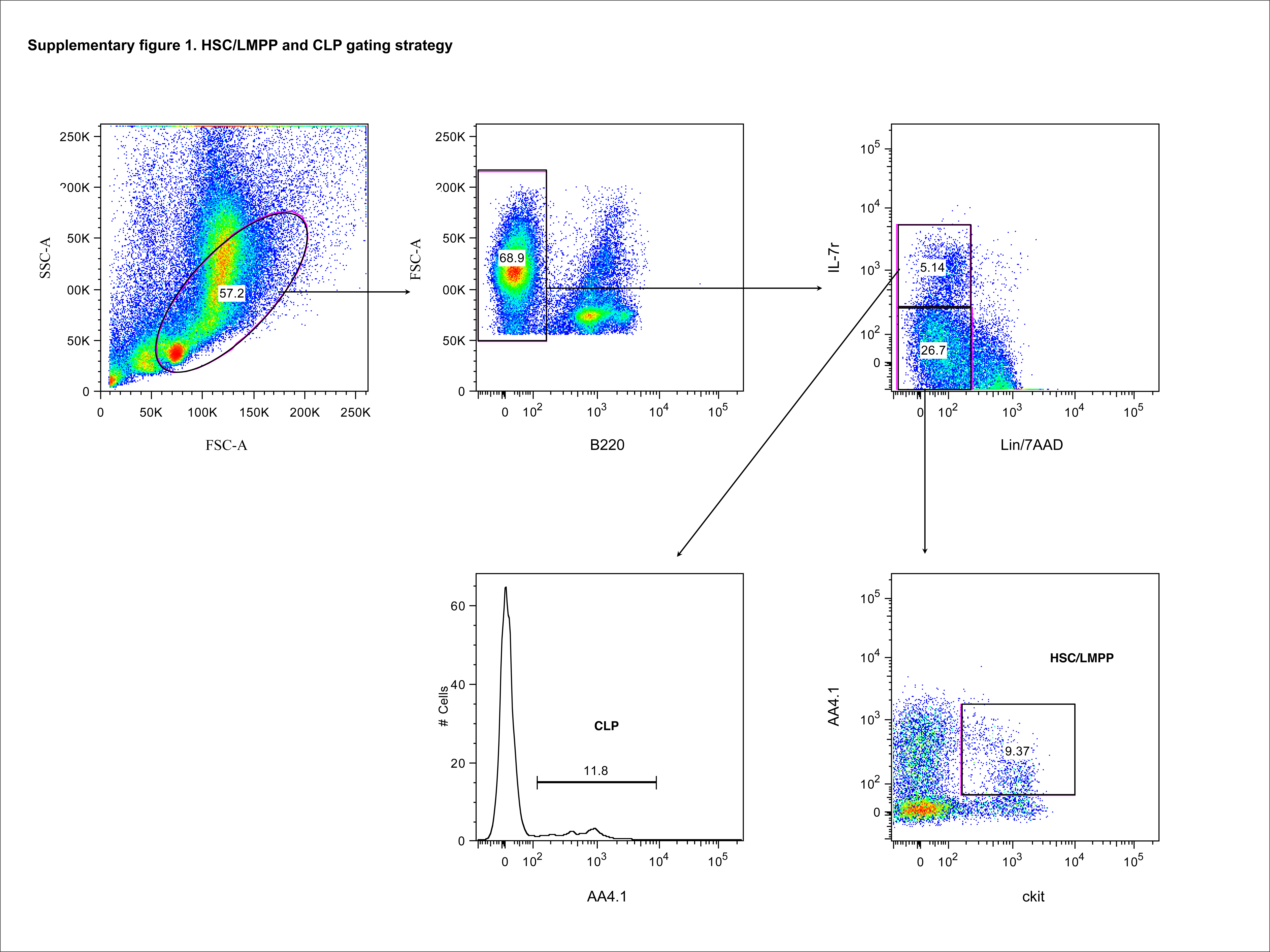

Supplement: Figure S1 — HSC/LMPP and CLP gating strategy. Representative plots obtained using bone marrow or spleen cells from uninfected mice stained for HSC/LMPP and CLP cells as described in table 1. (TIF) [file ppat.1002089.s001.tif]

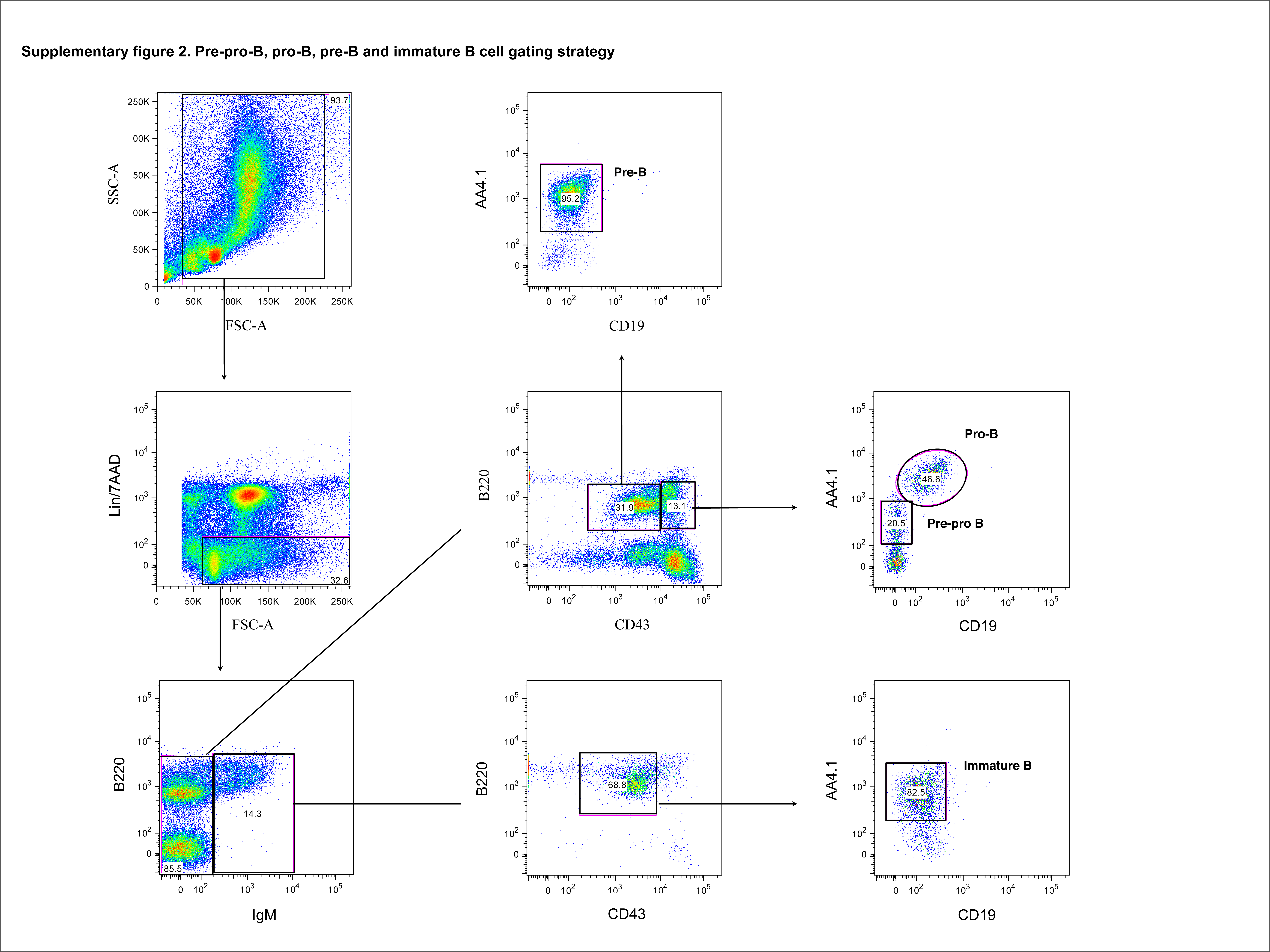

Supplement: Figure S2 — Developing and immature B cell gating strategy. Representative plots obtained using bone marrow or spleen cells from uninfected mice stained for pre-pro B, pro-B, pre-B and immature B cells as described in table 1. (TIF) [file ppat.1002089.s002.tif]

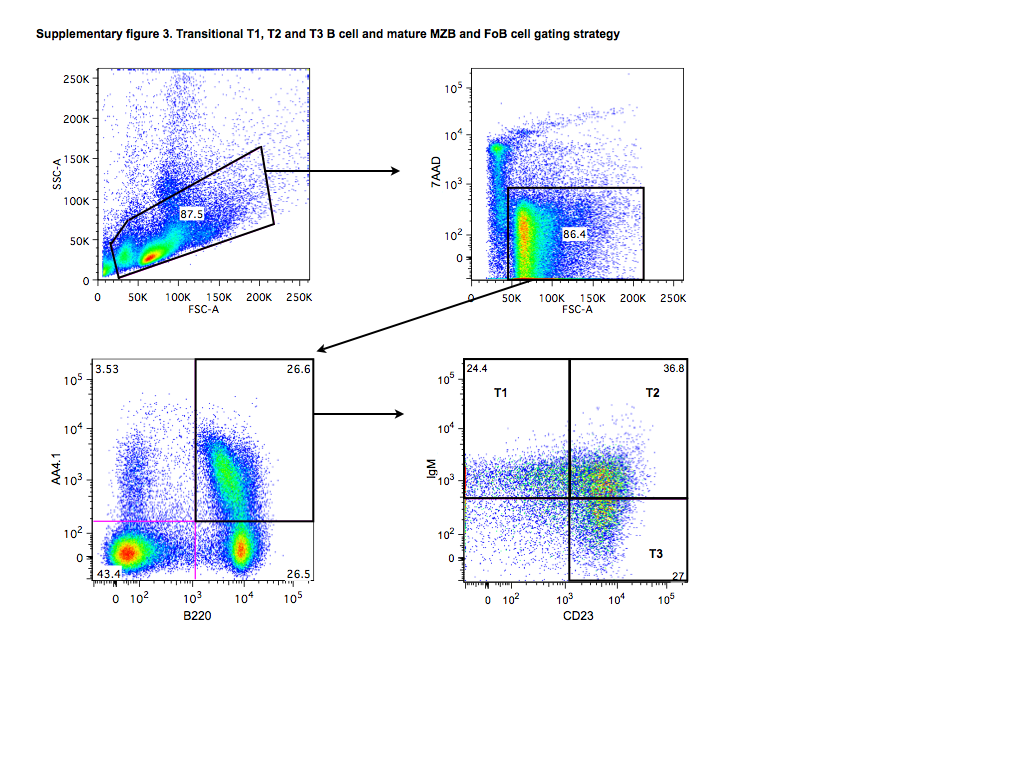

Supplement: Figure S3 — Transitional and splenic mature B2 B cell gating strategy. Representative plots obtained using spleen cells from uninfected mice stained for Transitional T1, T2 and T3 B cells and mature MZB and FoB cells as described in table 1. (TIF) [file ppat.1002089.s003.tif]

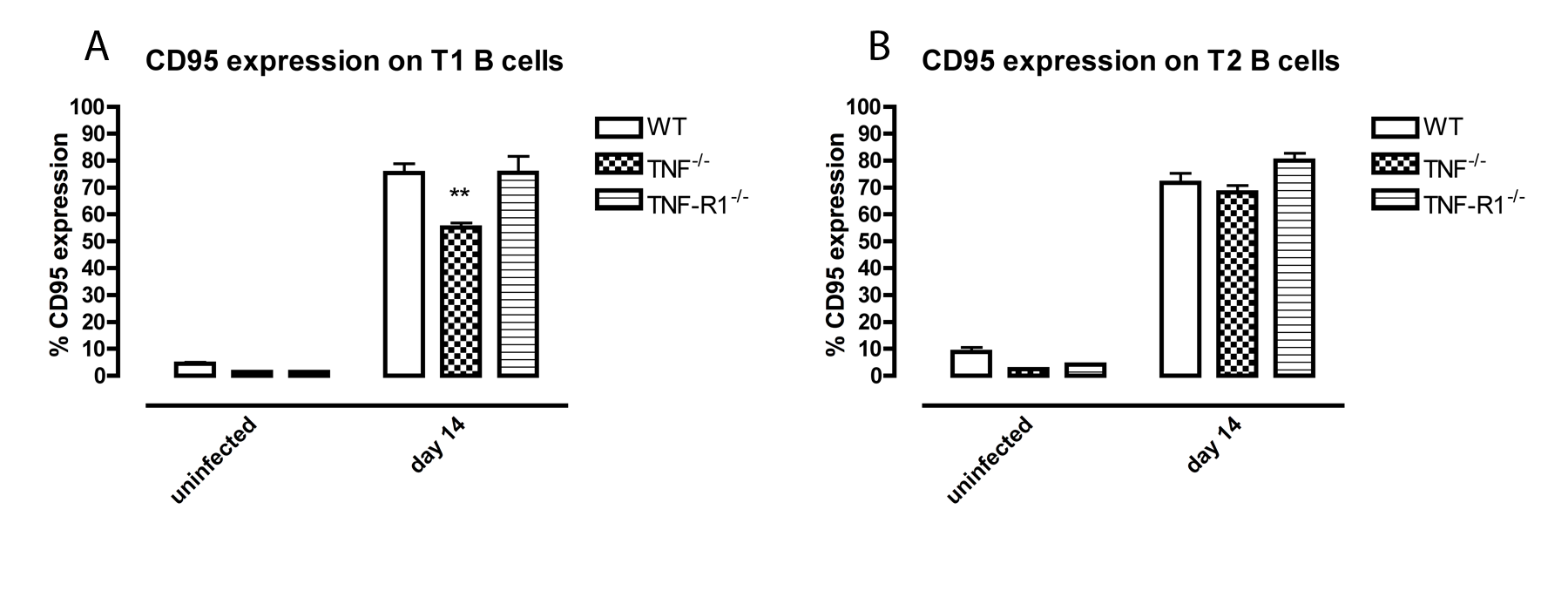

Supplement: Figure S4 — CD95 (Fas) expression on T1 and T2 transitional B cells in TNF−/− and TNF-R1−/− mice. (A) Percentage of CD95 expression on transitional T1 (left) and T2 (right) B cells in C57Bl/6 WT mice versus TNF− /− mice in uninfected controls and on day 14 pi. (B) Percentage of CD95 expression on transitional T1 (left) and T2 (right) B cells in C57Bl/6 WT mice versus TNF-R1− /− mice in uninfected controls and on day 14 pi. Data are represented as mean of three mice per group ± SEM. (TIF) [file ppat.1002089.s004.tif]

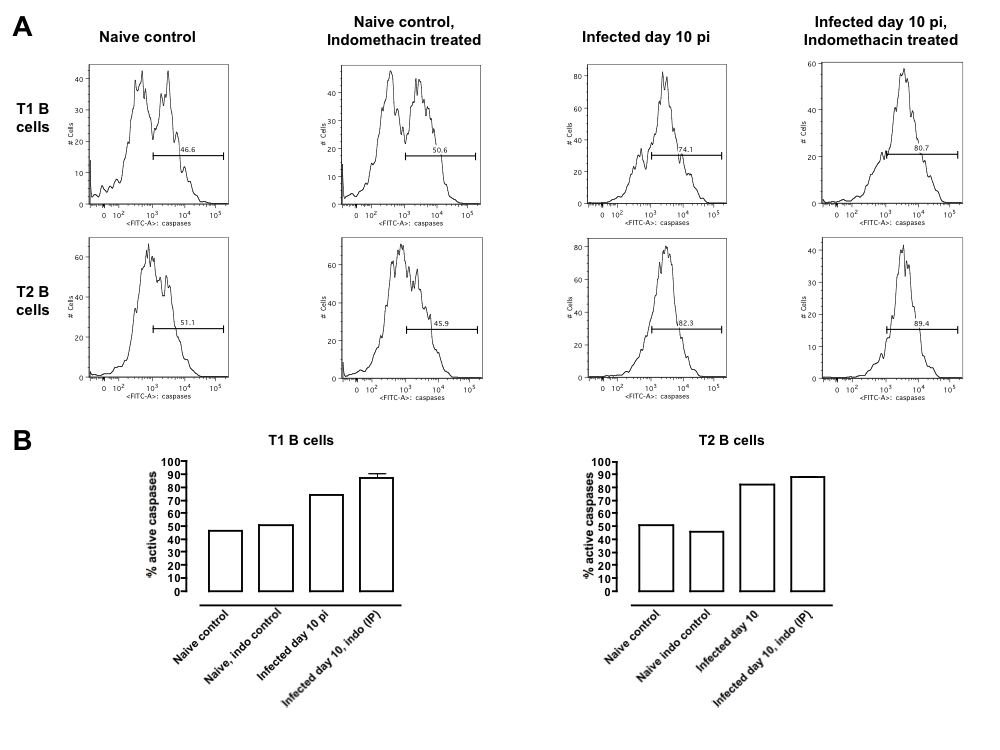

Supplement: Figure S5 — In vivo inhibition of the cyclooxygenase pathway during T. brucei infection using indomethacin, Indomethacin was administered to mice infected with T. brucei and uninfected control mice by daily i.p. injection and on day 10 of infection mice were sacrificed and an apoptosis assay was performed. (A) Representative histogram of the amount of active caspases inside T1 transitional B cells (upper panel) and T2 transitional B cells (lower panel). (B) Percentage of transitional T1 (left) and T2 (right) transitional B cells undergoing apoptosis. Data are represented as mean of 2 mice per control group and 3 mice per experimental group ± SEM. (TIF) [file ppat.1002089.s005.tif]

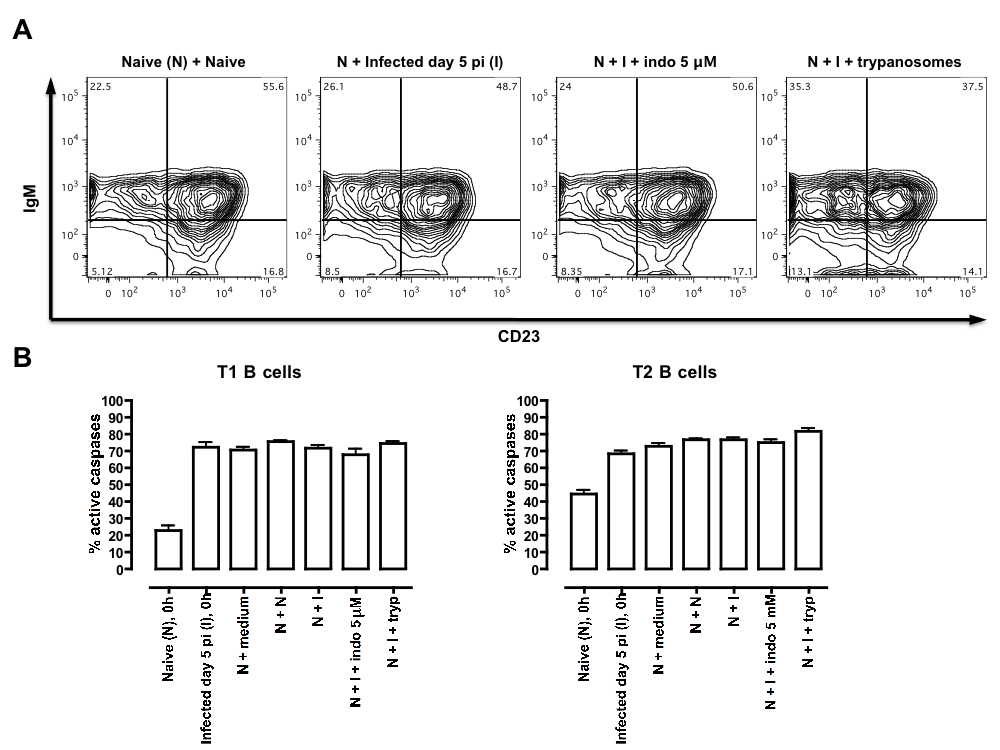

Supplement: Figure S6 — In vitro co-culture transwell system to investigate the contribution of the cyclooxygenase (COX) pathway in the induction of transitional B cell apoptosis. Total spleen cells from uninfected mice were co-cultured in a transwell system separated by a 0,4 µm polycarbonate transmembrane, with either medium or uninfected cells as a control or total spleen cells from mice 5 days post infection in the absence or presence of indomethacin (a nonsteroidal anti-inflammatory drug that inhibits COX activity) and/or trypanosomes. (A) Representative plots of T1 (AA4.1+ B220+ IgMhi CD23−) and T2 (AA4.1+ B220+ IgMhi CD23+) transitional B cells cultured under the different conditions. (B) Percentage of transitional B cells undergoing apoptosis in the different co-culture conditions. (TIF) [file ppat.1002089.s006.tif]

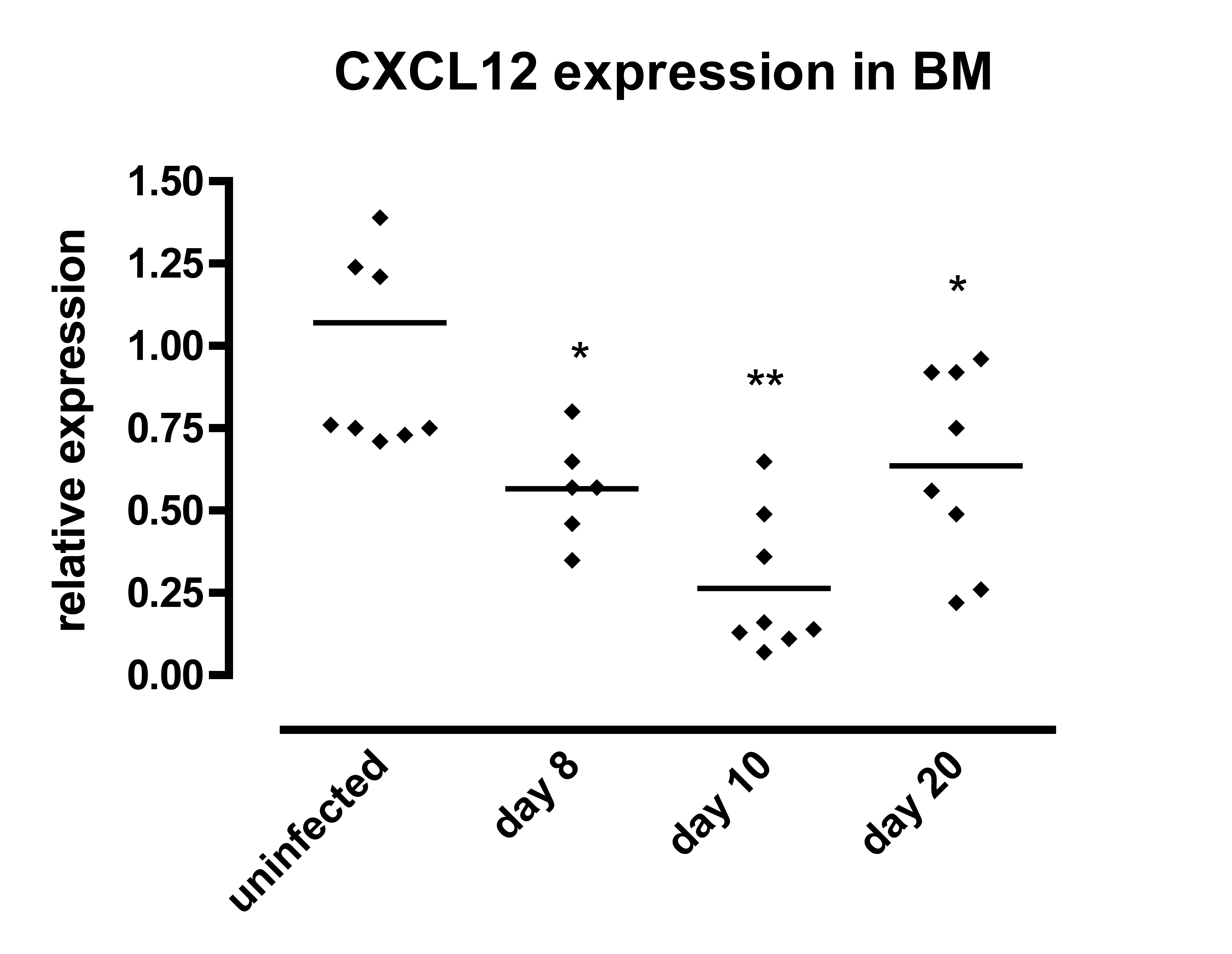

Supplement: Figure S8 — Bone marrow CXCL12 mRNA expression during T. brucei infection BM was isolated and amplified using intron-spanning primers specific for CXCL12 via Quantitative PCR. Data were normalized to GAPDH expression and are presented as relative expression compared to uninfected controls. Data are represented as mean of 6 or 8 mice ± SEM. (*) p<0,05, (**) p<0,01. (TIF) [file ppat.1002089.s008.tif]

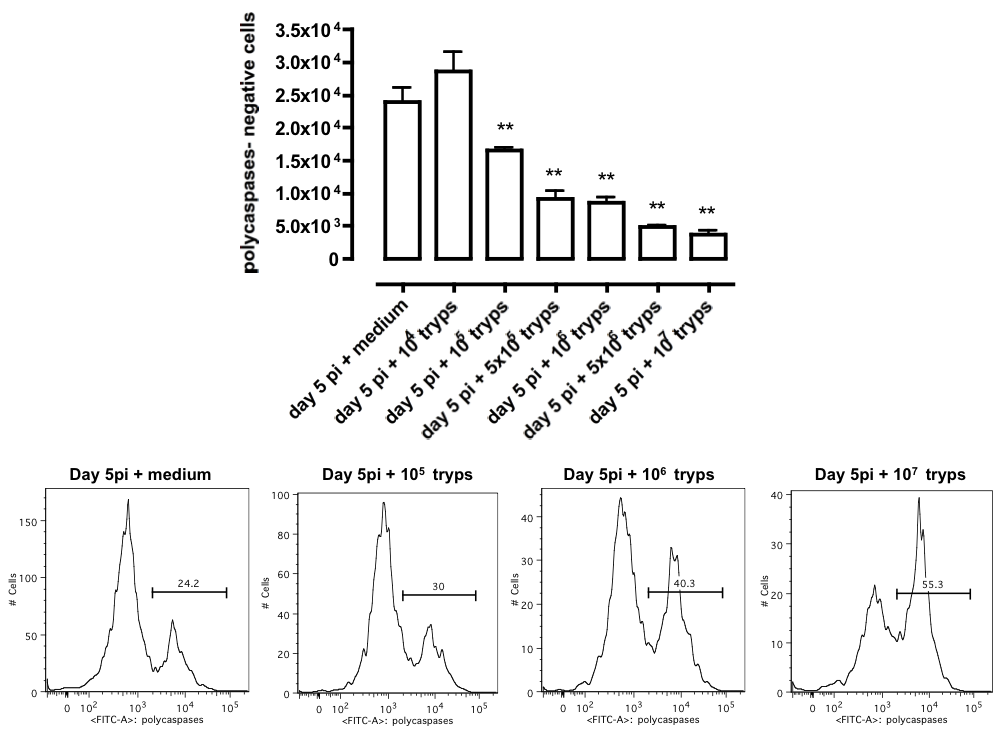

Supplement: Figure S9 — I n vitro co-culture of total spleen cells and trypanosomes. Total number of poly caspases-negative transitional B (T1 and T2) cells left after 20 h of co-culture of total spleen cells from uninfected mice with live bloodstream form trypanosomes or with medium only as control. Data are presented as mean of three mice per group ± SEM and three independent repeat experiments were performed (**) p<0,01. (TIF) [file ppat.1002089.s009.tif]
